# Parenting styles, feeding styles and food-related parenting practices in relation to toddlers’ eating styles: A cluster-analytic approach

**DOI:** 10.1371/journal.pone.0178149

**Published:** 2017-05-24

**Authors:** Klazine van der Horst, Ester F. C. Sleddens

**Affiliations:** 1 Nestlé Research Center, Institute of Nutritional Science, Lausanne, Switzerland; 2 Department of Health Promotion, NUTRIM School of Nutrition and Translational Research in Metabolism, Maastricht University Medical Center+, Maastricht, the Netherlands; Hospital Universitario de la Princesa, SPAIN

## Abstract

**Introduction:**

Toddlers’ eating behaviors are influenced by the way parents interact with their children. The objective of this study was to explore how five major constructs of general parenting behavior cluster in parents of toddlers. These parenting clusters were further explored to see how they differed in the use of feeding strategies (i.e. feeding styles and food parenting practices) and by reported child eating styles.

**Methods:**

An online survey with 1005 mothers/caregivers (legal guardians) with at least one child between 12 and 36 months old was conducted in the United States in 2012, assessing general parenting behavior, feeding style, food parenting practices and the child eating styles.

**Results:**

A three cluster solution of parenting style was found and clusters were labelled as overprotective/supervising, authoritarian, and authoritative. The clusters differed in terms of general parenting behaviors. Both overprotective and authoritative clusters showed high scores on structure, behavioral control, and nurturance. The overprotective cluster scored high on overprotection. The ‘authoritarian’ cluster showed lowest levels of nurturance, structure and behavioral control. Overprotective and authoritative parents showed very similar patterns in the use of food parenting practices, e.g. monitoring food intake, modeling, and promoting healthy food intake and availability at home. Overprotective parents also reported higher use of pressure to eat and involvement. Authoritarian parents reported high use of giving the child control over their food behaviors, emotion regulation, using food as a reward, and controlling food intake for weight control. Children’s eating styles did not largely vary by parenting cluster.

**Conclusion:**

This study showed that a relatively new parenting style of overprotection is relevant for children’s eating behaviors. Overprotective parents reported food parenting practices that are known to be beneficial for children’s food intake, such as modelling healthy food intake, as well as more unfavorable practices such as pressure. Longitudinal data on parenting practices and their relation to healthy eating in children is needed to inform communication and interventions for parents, reinforcing key feeding strategies which have positive effects on child eating behaviors and addressing parenting styles that have unintended negative effects.

## Introduction

The development of healthy eating behaviors in toddlers is often of concern to parents because many toddlers show certain difficulties with eating such as picky eating behaviors and/or neophobia [1]. Parents influence toddlers’ food intake through the foods they make available as well as through the way they interact with their toddlers. Previous reviews have suggested a relationship between particular parental feeding strategies and children’s energy intake, diet quality and body weight [2, 3]. Restrictive parenting practices were often associated with poorer child eating outcomes (e.g., the consumption of more unhealthy foods) [4–7]. Other practices parents use were found to be positively associated with child eating behaviors. For example, a positive association was found between parental modeling of healthy eating behaviors and child fruit and vegetable intake [8], and more covert control was found to be associated with less unhealthy snack intake and more fruit and vegetable intake in children [9]. Parents can use a wide range of potential practices or strategies to control their child's food intake and eating behavior [10]. It is important to identify which practices and strategies contribute positively to establishing healthy eating habits in children and which practices should be discouraged. It has been recommended to expand the focus from well-studied restrictive practices to include more positive practices such as modeling healthy eating and providing healthy food in the home, when examining the relationship between parental feeding strategies and child food intake [11]. This is particularly necessary when examining broader child-eating styles such as picky eating and emotional eating, because parent–child interactions are likely to involve a wide range of parental behaviors across a range of situations.

In the literature, two different types of parental feeding strategies exist: feeding styles and the aforementioned specific food parenting practices [12, 13]. The difference between the two is that feeding styles describe the more general parent-child interactions across food-related situations whereas food parenting practices include specific behaviors or rules parents use to control what, how much, or when their child eats, through, for example, pressure to eat vegetables, restricting foods, using foods as a reward or making foods available and accessible [14]. Feeding styles can be determined by a combination of the two underlying dimensions of demandingness and responsiveness. Demandingness refers to *how much* the parent encourages the child to eat in general e.g. saying to the child “Hurry up and eat your food” (vs. “eat your vegetables” as a practice). Responsiveness refers to *how* the parents encourage child eating, that is, in a responsive child-centered way e.g. arranging the food to make it more interesting and compliment the child for eating food, or in a non-responsive or adult-centered way (show disapproval for child not eating and physically struggle with child to get him/her to eat) [15, 16].

Additionally, it has been shown that non-food related parenting styles are also associated with children’s eating behavior [17]. These general parenting styles are a function of the parents’ attitudes, beliefs, and behaviors, and provide the socio-emotional context in which specific parenting practices are implemented [18], reflecting an approach to childrearing across situations and domains. Generally, three different parenting styles are defined based on Baumrind’s taxonomy of parenting styles: authoritarian, permissive, and authoritative [19]. Sometimes, a fourth parenting style is described, the neglectful or uninvolved parenting style. The four-fold typology of parenting is usually based on crossing the often used dimensions of responsiveness and demandingness [20], characterizing authoritative styles as parents who are both responsive and demanding, authoritarian styles as parents who are less responsive but highly demanding, permissive or indulgent styles as parents who provide a high level of responsiveness but are less demanding and neglectful or uninvolved styles as parents who show relatively low levels of both dimensions.

A wide array of instruments exists to assess parenting styles [17] making it difficult to compare findings between studies. Studies often measure multiple aspects of parenting, but do not examine how they co-occur. This study therefore used the recently developed Comprehensive General Parenting Questionnaire (CGPQ) [21] that aims to assess all major constructs of parenting to further explore which parenting styles can be identified and how these styles differ relative to food-related parenting practices and feeding styles used. The CGPQ measures well defined constructs such as nurturance, the degree to which parents are responsive to their child’s needs, showing interest in and spending time with the child, expressing affection and care, and structure, the degree to which parents help to organize the child environment, support the child to achieve goals and setting rules and boundaries to support the child. In this questionnaire, control was defined with three constructs: overprotection, behavioral control and coercive control. Overprotection is an understudied aspect in relation to eating behaviors; it is defined as excessive involvement of the parent or described as “helicopter parents” [22]. Behavioral control refers to parents supervising and managing child’s activities in a non-intrusive manner with clear expectations. Coercive control is a more intrusive manner of control, characterized by pressure, domination and discouragement of the child’s independence and individuality [21]. Using the CGPQ enables researchers to evaluate the effects of different parenting constructs and explore the presence and characteristics of different clusters of parenting.

Instead of examining a direct association between parenting and child eating behaviour, a limited number of studies has assessed if parents with different general parenting styles use different feeding strategies or so-called food parenting practices. In a recent review study by Collins et al. [23], authoritarian parenting was associated with pressuring a child to eat and using restrictive food parenting practices. Authoritative parenting was associated with parental monitoring of child food intake, whereas permissive parenting was inversely associated to parenting monitoring. Other studies report that authoritative feeding was positively associated with parental attempts to get the child to eat dairy, fruit, and vegetables [24], and lower use of pressure to eat by fathers only [25]. Based on two systematic reviews it can be concluded that children raised in authoritative households ate more healthily and authoritative parenting was associated with a healthy BMI [17, 26]. However, the effects of these generic parenting styles on weight-related behaviors were generally indirect and weak compared with the effects of more proximal behavior-specific parenting practices [27].

Studies examining the role of parenting styles and parenting practices in relation to child eating behavior have been limited. This study explores how the five major individual constructs in general parenting (nurturance, structure, behavioral control, coercive control, and overprotection) cluster in parents of toddlers. The hypothesis for this analyses is that the clusters would match the often used typology of authoritative, authoritarian, permissive and uninvolved parenting. A second aim of this study is to explore how these parenting clusters differ in the use of feeding styles, food parenting practices, and reported toddlers’ eating styles. Based on earlier studies, it was expected that authoritarian parents use more pressure and restriction, and that authoritative parents would use more monitoring, promoting healthy eating and less pressure [23]. Because the literature on toddler feeding is limited [28], a final objective of the study is to explore associations between parental feeding styles and food parenting practices with toddlers’ eating styles.

## Methods

### Respondents and procedure

A market research agency was contracted to conduct an online survey between August and October 2012, recruiting 1000 mothers/caregivers (legal guardians) with at least one child between 12 and 36 months old. Participating mothers/caregivers were at least 21 years of age and a specific quota was set to ensure that mothers/caregivers represented all larger regions from the USA (Northeast, Midwest, South, West). No other specific quotas were set. Participants were compensated with points according to the standard procedures of the online panel provider. The final number of participants in the study with completed questionnaires was N = 1005 (out of 2167 (46%)). The mean length of time to complete the online questionnaire was 26 minutes. Most of the participants were married (83%), white/Caucasian (81%), with a mean age of 31.8 years old (range 21 to 69). Almost half of the participants reported being employed full/part time (including self-employment; 46%), and almost 60% had graduated from college, technical school or higher. The mean age of the toddlers was 24.6 months (range 12–36 months), and 528 (53%) of them were boys and 477 were girls. For 31% of the participants this was the only child under 18 years-old in the household (Table 1). Participants did not have to provide written or verbal consent for this part of the study. They were invited to answer the questionnaire and by filling out the questionnaire, respondents were agreeing to participate. Participation was voluntary and study participation could be stopped at any time. Mothers or legal guardians answered the questionnaires, therefore children were not involved in the study. The study protocol was approved by the Copernicus Group IRB (NES1-12-166).

**Table 1:** Participant characteristics (N = 1005).

Description	n	%
Child gender		
Boy	528	52.5
Girl	477	47.5
Mothers ethnicity		
Black/African American	73	7.3
White/Caucasian	813	80.9
Asian/Pacific Islander	51	5.1
Native American/Alaskan Native	7	0.7
Multiracial/Other	51	5.1
Prefer not to answer	10	1.0
Mothers education		
Some high school	11	1.1
Graduated from high school	155	15.4
Some college or technical school	244	24.3
Graduated community college or technical school	112	11.1
Graduated college	336	33.4
Post graduate work	50	5.0
Advanced degree	93	9.3
Prefer not to answer	4	0.4
Employment status		
Yes, full time, outside of home	285	28.4
Yes, part time, outside of home	121	12.0
Yes, full time self-employed	23	2.3
Yes, part time self-employed	34	3.4
Not employed	534	53.1
Prefer not to answer	8	0.8
Household total annual income		
Under $20,000	96	9.6
$20,000–$39,999	233	23.2
$40,000–$59,999	225	22.4
$60,000–$79,999	184	18.3
$80,000–$99,999	113	11.2
$100,000 or over	116	11.5
Prefer not to answer	38	3.8
Number of children <18 in household		
1 child	313	31.1
2 children	405	40.3
3 children	189	18.8
4 children	68	6.8
5 children	18	1.8
6 or more children	12	1.2
Marital staus		
Married	833	82.9
Single	78	7.8
Widowed	1	0.1
Divorced	24	2.4
Separated	15	1.5
Domestic partner	52	5.2
Prefer not to answer	2	0.2

### Measures

Validated instruments were used to measure general parenting, feeding styles, food parenting practices, and child eating styles: the Comprehensive General Parenting Questionnaire (CGPQ) [21], the Caregiver’s Feeding Style Questionnaire (CFSQ) [15, 16], the Comprehensive Feeding Practices Questionnaire (CFPQ)[29], and the Children’s Eating Behavior Questionnaire (CEBQ) [30].

#### General parenting

Five parenting constructs were assessed with the CGPQ [21]: nurturance, structure, behavioral control, coercive control, and overprotection. Nurturance represents the degree to which parents are supportive and responsive to the child’s needs, support their child’s autonomy, use praise and express affection and care towards their child. Structure represents the degree to which parents help the child to organize activities, help them to achieve goals and are having consistent rules and boundaries for child behavior. Behavioral control refers to supervising, being clear on expectations for behavior, using non-intrusive discipline by for instance explaining misbehaviors, that allow the child to have enough space to develop independence and autonomy. Coercive control refers to pressure, intrusion, domination and discouragement of the child’s independence and individuality. Overprotection refers to being too involved with the child, and excessively monitoring of the child’s behavior.

The original CGPQ was developed for parents of children aged 5 to 13 [21]. The CGPQ was modified for use with parents of toddlers, together with the developers of the CGPQ. The final CGPQ is presented in the S1 Table. The questionnaire items were reviewed, and wording of several questions was changed and the excessive monitoring sub-scale was removed. This sub-scale was considered to be less relevant because parental monitoring levels for toddlers are generally high. To cover parent involvement properly, two items were added to the excessive involvement sub-scale. The modified questionnaire was pre-tested by conducting cognitive interviews with eight mothers of toddlers 12–36 months old to find out if the mothers considered the parenting style questions (culturally) appropriate, clear and relevant for the age of their toddlers. Feedback from the pre-test was used to develop an 81-item version that was tested in a pilot study. In total, 332 mothers that were listed in a panel database answered the online pilot questionnaire: 79 mothers with a child between 12–17 months, 85 with a child between 18–23 months, 93 with a child between 24–30 months, and 74 with a child between 31–36 months. One record was deleted because the reported child age was 55 months. After pilot testing, another 8 items were dropped because more than 15% of the mothers indicated that these items were not relevant for their child’s age. Four items were dropped following Confirmatory Factor Analyses on the resulting 73 items due to low factor loadings and following discussions among the developers of the CGPQ. The final 69-item questionnaire was used in this study (S1 Table). The nurturance scale consisted of 18 items with a Cronbach’s alpha of 0.92, the structure scale (17 items) had a Cronbach’s alpha of 0.83, the behavioral control scale (13 items) had a Cronbach’s alpha of 0.91, the coercive control scale (14 items) had a Cronbach’s alpha of 0.85 and the overprotection scale (7 items) had a Cronbach’s alpha of 0.65. Parents were asked to rate the parenting items on a five-point Likert scale from 1 (Strongly disagree) to 5 (Strongly agree).

#### Feeding style

Feeding style was assessed with the CFSQ [15, 16]. Items from the CFSQ measure patterns of feeding along two dimensions (i.e. parental demandingness and responsiveness regarding their child’s eating). The demandingness scale consisted of 19 items (e.g. “How often during the dinner meal do you physically struggle with your child to get him/her to eat (for example, physically putting the child in the chair so he or she will eat)?”), with a Cronbach’s alpha of 0.90 The responsiveness scale consisted of seven items (e.g. “How often do you encourage your child to eat by arranging the food to make it more interesting (for example, making smiley faces on the pancakes)?”), with a Cronbach’s alpha of 0.72. Considerable support was provided for the validity of the CFPQ [29], and Cronbach’s alphas of most of the scales were moderate to high in the original study of Hughes et al.[16]. Parents were asked to rate the feeding style items on a five-point Likert scale from 1 (Never) to 5 (Always). To score demandingness, a total mean score was calculated across all items; to score responsiveness, a ratio of child-centered items over the total score was calculated conform the original study.

#### Food parenting practices

The CFPQ [29] was used to capture a broad range of behaviors that parents might engage in when feeding their children. This scale was validated with parents of children aged 18 months–8 years). Good test-retest reliability was established and validity through associations with other measures of child feeding and general parenting were presented in the original study of Musher-Eizenman and Holub [29]. The questionnaire assesses the following parenting practices: allowing the child control over his/her eating behaviors and parent-child feeding interactions, e.g. “Do you let your child eat whatever he/she wants?” (Child control, 5 items, Cronbach’s α = 0.70), use of foods to regulate the child emotions, e.g. “Do you give the child something to eat or drink if he/she is upset even if you think s/he is not hungry?” (Emotion regulation, 3 items, Cronbach’s α = 0.83), encouragement to eat well-balanced and healthy meals, e.g. “I encourage my child to try new foods” (Encourage balance and variety, 4 items, Cronbach’s α = 0.82), making healthy foods available in the home, e.g. “Most of the food I keep in the house is healthy” (Environment, 4 items, Cronbach’s α = 0.73), use of food a reward for child behavior, e.g. “I offer sweets (candy, ice cream, cake, pastries) to my child as a reward for good behavior” (Food as reward, 3 items, Cronbach’s α = 0.73), encouragement of the child’s involvement in meal planning and preparation, e.g. I encourage my child to participate in grocery shopping” (Involvement, 3 items, Cronbach’s α = 0.74), parents’ own demonstration of healthy eating to the child, e.g. “I model healthy eating for my child by eating healthy foods myself” (Modeling, 4 items, Cronbach’s α = 0.86), tracking of the child’s less healthy food intake, e.g. “How much do you keep track of the sweets (candy, ice cream, cake, pastries) that your child eats” (Monitoring, 4 items, Cronbach’s α = 0.91), pressure to let the child eat more food at meals, e.g. “My child should always eat all the food on his/her plate” (Pressure, 4 items, Cronbach’s α = 0.70), controlling food intake to limit less healthy foods and sweets, e.g. “If I did not guide or regulate my child’ eating, he/she would eat too much of his/her favorite foods” (Restriction for health, 4 items, Cronbach’s α = 0.78) and controlling the child’s food intake to decrease or maintain the child’s weight, e.g. “I give my child small helpings at meals to control his/her weight (Restriction for weight control, 8 items, Cronbach’s α = 0.86). The last practice, teaching about nutrition, was not included in our questionnaire as the items were of less relevance due to the young age of the children. Mean scores were computed for subscales.

#### Child eating styles

With the Child Eating Behaver Questionnaire (CEBQ) children’s appetites are characterized by measuring eight factors which can be divided into two main dimensions: food approach and food avoidance. These concepts can be used to describe movements toward or away from food as well as by using the individual factors to describe children’s eating behaviors [30–33].

The food approach subscales consisted of ‘food responsiveness’ e.g. “If allowed to, my child would eat too much” (5 items, Cronbach’s α = 0.82), ‘enjoyment of food’ e.g. “My child is interested in food” (4 items, Cronbach’s α = 0.87), ‘emotional overeating’ e.g. “My child eats more when worried” (4 items, Cronbach’s α = 0.80), and ‘desire to drink’ e.g. “My child is always asking for a drink” (3 items, Cronbach’s α = 0.85). The food avoidant subscales consisted of ‘satiety responsiveness’ e.g. “My child cannot eat a meal if he/she has had a snack just before” (5 items, Cronbach’s α = 0.73), ‘slowness in eating’ e.g. “My child take more than 30 minutes to finish a meal” (4 items, Cronbach’s α = 0.70), ‘emotional undereating’ e.g. “My child eats less when angry” (4 items, Cronbach’s α = 0.64), and ‘food fussiness’ e.g. “My child refuses new foods at first” (6 items, Cronbach’s α = 0.88). The scales were shown to have good internal consistency in the original study conducted by Wardle et al.[30]. Parents were asked to rate the child's eating style items on a five-point Likert scale from 1 (Never) to 5 (Always). Mean scores were computed for each subscale and as well for the combined scales of food approach behaviors (Cronbach’s α = 0.60) and food avoidant behaviors (Cronbach’s α = 0.71).

### Data analyses

To examine if parents could be grouped based on sharing similar patterns of parenting style, a cluster analysis was performed based on the five parenting constructs. Cluster analysis assigns participants to groups in a way that participants within groups are as similar as possible and participants between groups are as dissimilar as possible. First, a two-step clustering procedure, suitable for relatively large sample sizes was performed on standardized scores of the parenting constructs. A three cluster solution was further examined with K-means clustering. This three-cluster solution was found to be adequate and meaningful regarding the different patterns found. The three clusters were compared with ANOVA on continuous demographic variables, feeding style dimensions, food parenting practices and all child eating styles subscales. All eating styles subscales were used because it is of interest to have a more detailed look at how the specific eating styles differ according to parenting clusters. Bonferroni correction for multiple testing was conducted by dividing the desired alpha-level (0.05) by the number of comparisons [25]. This resulted in a p-value of 0.002 for determining significance of the ANOVA comparisons. Significant F-tests were followed by the examination of contrasts using Tukey HSD tests. When the assumption of homogeneity of variance was violated, the Welch F procedure was applied followed by examination of contrasts using the Games-Howell post hoc procedure. For the categorical demographic variables, Chi-Square tests were performed. In order to examine which parental feeding strategies (i.e. feeding styles and food parenting practices) were associated with healthy child eating behaviors, child eating style was divided into food approach styles (emotional overeating, food responsiveness, enjoyment of food and desire to drink) and children’s food avoidant styles (emotional undereating, food fussiness, satiety responsiveness and slowness in eating). Past research has shown that high scores on food approach have been associated with higher weight status while high scores on food avoidance have been associated with a lower weight status among preschoolers [34–36]. Two linear regression analyses were performed. First, food approach and avoidant styles were used as dependent variables in the model, with the feeding styles and food parenting practices as independent variables. Demographic variables (caregivers’ age and education, household income, ethnicity, child gender, child age in months) were included as covariates. Data were analyzed with IBM SPSS Statistics 21.

## Results

### Cluster analysis of parenting styles

**Fig 1:**
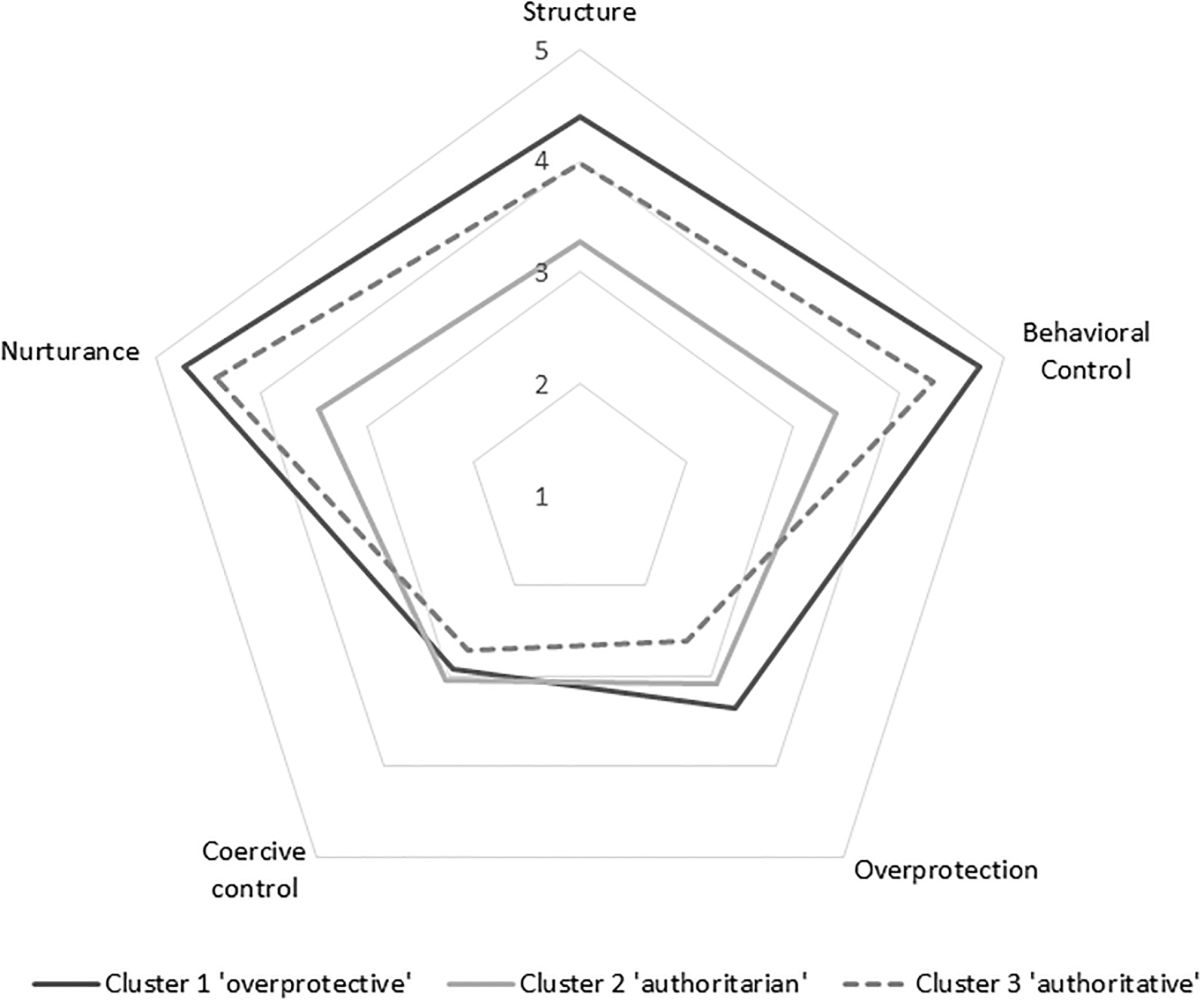
Representation of the three clusters for the main constructs of parenting style^a^. ^a^ Mean scores (standard deviation) on the main constructs for the 3 clusters (cluster 1 = C1, cluster 2 = C2, cluster 3 = C3) were as follows: Structure: C1 = 4.39 (0.30) C2 = 3.28 (0.28) C3 = 3.97 (0.33); Behavioral Control: C1 = 4.76 (0.22), C2 = 3.41 (0.41), C3 = 4.32 (.31); Overprotection: C1 = 3.36 (.57), C2 = 3.08 (.43), C3 = 2.61 (.62); Coercive control: C1 = 2.93 (.72), C2 = 3.05 (.43), C3 = 2.71 (.62); Nurturance: C1 = 4.73 (.19), C2 = 3.47 (.37), C3 = 4.43 (.29).

Fig 1 illustrates the results of the three cluster solution. These three clusters were labelled as follows: cluster 1: overprotective/supervising; cluster 2: authoritarian; and cluster 3: authoritative. The ‘overprotective/supervising’ cluster was characterized by the highest mean values on four of the five parenting constructs: nurturance, structure, behavioral control, and overprotection. The ‘authoritative’ cluster was also characterized by high values on nurturance, structure and behavioral control, but lower values on coercive control and overprotection. Compared to the other two clusters, the ‘authoritarian’ cluster showed low levels of nurturance, structure and behavioral control. n Significant differences between clusters were found for ethnicity, responsiveness, most of the food parenting practices, enjoyment of food and fussiness (Table 2). More Caucasian caregivers were allocated to the authoritative cluster (87.4%) than in the overprotective (77.2%) or authoritarian (78.4%) clusters. Responsiveness was found to be higher in overprotective (M = 1.23) and authoritative (M = 1.20) mothers than in authoritarian mothers (M = 1.09), while demandingness did not differ between parenting clusters.

**Table 2:** Comparison of the three clusters for feeding style, food parenting practices and child eating styles.

	Overprotective supervising N = 464	Authoritarian N = 111	Authoritative N = 430	F/Chi2*
**Demographics**				
Cargivers age	31.7	31.3	32.0	1.1
Caregivers education	4.08	4.00	4.22	1.4
Household income	3.32	3.19	3.40	0.9
WIC participation (% yes within cluster)	27.6%	26.1%	23.9%	1.6
Ethnicity (% Caucasian)	77.2%	78.4%	87.4%	**16.0**
Child gender (% boys)	54.5%	49.5%	51.2%	1.5
Child age (months)	24.05	25.05	25.17	3.0
**Feeding Style**				
Demandingness	2.73	2.68	2.61	4.5
Responsiveness	1.23^b^	1.09^a^	1.20^b^	**41.9**
**Food parenting Practices**				
Child control	2.52^a^	2.87^b^	2.53^a^	**14.5**
Emotion Regulation	1.98^a^	2.71^b^	2.01^a^	**49.1**
Encourage balance and variety	4.69^b^	3.42^a^	4.40^b^	**228.3**
Environment	4.10^c^	3.22^a^	3.82^b^	**124.8**
Food as reward	2.53^a^	2.94^b^	2.39^a^	**24.3**
Involvement	3.60^b^	3.11^a^	3.20^a^	**30.4**
Modeling	4.36^c^	3.21^a^	3.97^b^	**151.1**
Monitoring	4.49^c^	3.35^a^	4.18^b^	**115.5**
Pressure	3.07^b^	3.02^b^	2.77^a^	**13.7**
Restriction for Health	3.13	3.11	3.10	0.08
Restriction for weight control	2.07^b^	2.77^c^	1.89^a^	**83.9**
**Child Eating Style**				
Emotional overeating	1.83	2.08	1.81	6.1
Emotional undereating	2.84	2.84	2.88	0.3
Desire to drink	3.49	3.27	3.33	4.5
Enjoyment of food	3.98^c^	3.49^a^	3.79^b^	**23.5**
Food responsiveness	2.61	2.65	2.51	2.7
Fussiness	2.57^a^	2.9^b^	2.71^b^	**10.1**
Slowness in eating	2.89	2.97	2.92	0.6
Satiety responsiveness	2.95	2.95	2.99	0.5

* Significant factors are indicated in bold. Significance for the ANOVA tests was set at p <.002 after Bonferroni correction for mulitple testing. In case the assumption of homogeneity of variance was violated, the Welch F procedure was conducted.

^a,b,c^ for significant difference (p <.05) between clusters with post hoc Tukey HSD or Games-Howell procedure. If a parenting practice has a similar superscript letter in two clusters this indicates a nonsignificant difference, if a parenting practice has different superscript letters in two clusters, this indicates a significant difference.

### Parenting practices and eating styles by cluster

All food parenting practices, except restriction for health, significantly differed between the parenting style clusters. Overprotective and authoritative parents showed very similar patterns in the use of food parenting practices with higher scores for keeping track of child’s intake of less healthy foods (monitoring), demonstrating healthy eating behaviors (modeling), promoting well-balanced food intake, including the consumption of varied foods and healthy food choices and making healthy foods available in the home. Overprotective parents also showed a higher level of encouraging the child’s involvement in meal planning and preparation (involvement) (M = 3.60), and pressure to eat (M = 3.07) while authoritative parents showed a slightly lower use of pressure to eat (M = 2.77). Authoritarian parents showed a different pattern in the use of food parenting practices with higher scores on controlling the child’s food intake with the purpose of decreasing or maintaining the child’s weight (M = 2.77), the use of food as a reward for child behavior (M = 2.94), the use of food to regulate the child’s emotional states (M = 2.71).

Children’s eating styles did not largely vary by parenting cluster. Overprotective parents reported lower levels of food fussiness in their toddlers. Enjoyment of food was reported to be significantly lower in children with parents in the authoritarian cluster. No significant differences were found for emotional undereating, emotional overeating, food responsiveness, desire to drink, slowness in eating and satiety responsiveness.

### Associations between feeding strategies and child eating styles

Parental feeding strategies were associated with parent-reported child food approach behaviors and food avoidance behaviors (see Table 3). The food approach regression model was R^2^ =.241, and the food avoidance model was R^2^ =.410. Caregivers reporting higher levels of responsiveness, emotion regulation, encouragement of balance and variety, food as a reward, involvement and restriction for health, were more likely to report higher levels of child food approach behaviors. Caregivers reporting lower levels of child control, reported higher levels of child food approach behaviors. Caregivers reporting higher levels of demandingness, child control, and restriction for health were more likely to report higher levels of child food avoidance behaviors. Caregivers reporting lower levels of responsiveness, food as reward, involvement, pressure and restriction for weight control were more likely to report higher levels of child food avoidance behaviors. Caregivers reported fewer food approach behaviors and more food avoidance behaviors by increasing toddlers’ age.

**Table 3:** Results of linear regression analyses with parental feeding strategies as independent and eating styles as dependent variables^a^.

	Food approach behaviors		Food avoidance behaviors	
	Beta^b^	P-Value	Beta^b^	P-Value
Caregivers’ age	-.073	.021	-0.47	.097
Caregivers’ education	.047	.159	0.27	.360
Household income	.009	.792	.017	.577
Ethnicity (white/Caucasian)	-.034	.260	-.004	.868
Childs’ sex (girls)	-.058	.045	.000	.988
Child age (months)	-.077	.014	0.64	.020
Demandingness	-.014	.727	.536	.000
Responsiveness	.136	.000	-.112	.001
Child control	-.135	.000	.155	.000
Emotion regulation	.312	.000	.003	.927
Encourage balance and variety	.094	.026	-.067	.072
Pressure	.044	.233	-.147	.000
Monitoring	-.022	.541	-.014	.656
Modelling	-.016	.700	-.002	.952
Food as reward	.083	.029	-.087	.010
Involvement	.072	.027	-.091	.002
Environment	-.055	.163	.050	.156
Restriction for health	.166	.000	.127	.000
Restriction for weight control	.068	.064	-.079	.018

^a^ The model fit for the food approach regression model was R^2^ =.241, and for the food avoidance model R^2^ =.410.

^b^ Standardized regression coefficients from the linear regression analysis

## Discussion

The first topic that was explored in this study was how the five major individual constructs of general parenting behavior would cluster in parents of toddlers, to see if this matched often used typology of authoritative, authoritarian, permissive and uninvolved parenting. The analysis revealed three clusters we labelled overprotective/supervising, authoritarian and authoritative. In this sample the authoritarian cluster referred to lower levels of nurturance, structure (organizing child activities, help in achieving goals, consistent rules) and behavior control (supervising, clear expectations, explaining misbehaviors). Higher scores on these constructs referred to a positive or so-called authoritative parenting style in which the child needs are accounted for and parents give structure to the environment of the child to support them and setting clear expectations [21]. Both parenting styles were in line with existing research and the parenting style concept as proposed in the parenting literature [18–20]. In addition, we found one cluster with high levels of all five constructs of parenting, including overprotection. Overprotection or a phenomenon that is popularly referred to as helicopter parenting [22], refers to checking frequently where the child is and what the child is doing, more than is considered appropriate for the child’s age and the risks to which the child is exposed [37]. This is an understudied aspect of parent control [21] and this parenting style is not mentioned in the parenting style literature from the 1980s. It might be that forty years ago this parenting style was not yet common, while the fourth common parenting style of being uninvolved or neglective was not found in our study. This parenting style could have been more relevant when this literature was developed. The concept of overprotective parenting has received increasing public attention and there are suggestions that the prevalence of overprotective parenting has increased over the pasted two decades [22, 37]. Overprotection is considered to negatively impact child development by interfering with the development of child autonomy and it has been negatively associated with parental autonomy granting, school engagement [22] and physical activity in children aged 7–12 [38], and positively associated with overweight in 10–11 years old children [37]. The overprotective parents were characterized by having the highest levels of nurturance, structure and behavioral control which are all known characteristics of this parenting type [37, 38]. We found that overprotective and authoritative parents showed very similar patterns in parenting practices. Overprotective parents reported even higher use of monitoring intake of less healthy foods and making healthy foods available in the home and encouraging balance and variety than authoritative parents, practices that are beneficial for children’s eating styles [8, 39]. However, overprotective parents also reported higher use of pressure and restriction which is more similar to practices often reported in authoritarian parents [23] and which in most studies have a negative association with child eating [4, 5, 7]. A former study reported a significant positive association between the overprotective parenting construct and child Body Mass Index (BMI] [21], which might indicate that the overprotective parenting might have less favorable weight status outcomes in children due to the controlling practices and lower levels of physical activity [38].

Children’s eating styles did not vary much by parenting cluster. Only small differences were found for enjoyment of food, with the highest scores in overprotective and authoritative parents and for food fussiness with the lowest level reported for children with overprotective parents. Food fussiness and eating enjoyment are factors that are correlated with each other and it is therefore not surprising to find both high eating enjoyment and lower fussiness in children of overprotective parents [40]. High scores on food approach (i.e. eating enjoyment) have been associated with higher weight status while high scores on food avoidance (i.e. fussiness) have been associated with a lower weight status among preschoolers [34–36]. This might explain the association between overprotective parenting and overweight in children 10–11 years old [37] if eating styles mediate the associations between parenting style and body weight [41]. Because parenting style clusters are a more distal factor compared to specific parenting practices, the results confirm other studies that show weak results for the association of parenting styles with weight-related behaviors [17, 27]. Stronger correlations were found for specific parenting practices and child eating styles. Responsiveness, child control, food as reward, and involvement were found to be associated with both food approach and avoidance behaviors in opposite directions. A number of studies have found positive associations between parental restriction and food approach behaviors and child weight status [42–44]. Similar to other studies, we found a negative association between pressure to eat and food avoidance [32, 35, 40, 43].

There are several limitations to take into account when interpreting the results of the study. The cross-sectional design of this study prevents interpretations involving the interaction between parents and children [45–47]. There are limited studies that approach the bi-directional relationship between parenting behaviors and child eating or weight status with a longitudinal design [45–47]. These studies provide support for a child-responsive model in which parents tend to adapt their controlling strategies in response to their child’s BMI rather than the reverse [45–47], or at least a bidirectional relation [45]. Overt control which involves limiting the child's intake of unhealthy foods in a way that can be perceived by the child, was found to be a non-reactive practice that occurred independently of child weight but, when applied, influenced it negatively [45]. In addition, the study population included mainly mothers from white/Caucasian ethnic background, which made it impossible to examine the potential role of fathers and (in)consistencies in parenting between parents and ethnic background on parenting practices. In the current study dietary or weight variables were not assessed and the association between parenting and food intake or BMI could not be explored.

The cluster analytic approach was a strength that allowed us to assess the contribution of all five parenting constructs at the same time for better differentiation among parenting styles. As such, different combinations of the constructs could be used to characterize different clusters of parenting corresponding to the wide-spread use of Baumrind’s classic parenting typology. Another strength of this study is that the questionnaires were successfully modified and applied to young children. Previous parenting research in the food domain has mainly focused on primary school-aged children. However, parents have a large influence and control over the child eating habits, especially in the toddler period, when children are still learning to eat new foods, learning table manners and often experience picky eating behaviors. It is important that healthy eating habits are learned early in life as they have the tendency to persist into adolescence and adulthood [48].

## Conclusion

This study is one of the first to evaluate various types and levels of parental influences on child eating styles. In addition to authoritarian and authoritative parenting styles, a relatively new parenting style of overprotection is be relevant for children’s eating behaviors. Overprotective and authoritative parents showed very similar patterns in parenting practices such as monitoring unhealthy food intake and making healthy foods available, which are often found to be associated with healthy food intake in children. However, overprotective parents also reported higher use of pressure and restriction for weight control, similar to authoritarian parents. These more authoritarian practices are often found to have a negative association with children’s food intake. Future research could explore if overprotective parenting is also present among parents with children of various ages and from different cultural or socio-economic backgrounds. The relationship between overprotective parenting and associations with child eating and weight could also be explored. Longitudinal data on parenting practices and their relation to healthy eating in children is needed to inform communication and interventions for parents, reinforcing key feeding strategies which have positive effects on child eating behaviors and addressing parenting styles that have unintended negative effects.

## Supporting information

S1 Table Comprehensive General Parenting Questionnaire (caregivers of 1 to 4 year olds)^a^. ^a^ Based on Sleddens, O’Connor, Watson, Hughes, Power, Thijs, De Vries, & Kremers. Development of the Comprehensive General Parenting Questionnaire for caregivers of 5–13 year olds International Journal of Behavioral Nutrition and Physical Activity 2014, 11:15. CGPQ adapted to caregivers of 1–4 year olds by Ester Sleddens, Tom Power, Teresia O’Connor, Sheryl Hughes and Stef Kremers.
(DOCX)

S1 Datafile (XLSX)
