# Supplementary material for: Parenting styles, feeding styles and food-related parenting practices in relation to toddlers’ eating styles: A cluster-analytic approach
Source: PLoS One. 2017 May 24;12(5):e0178149. doi: 10.1371/journal.pone.0178149 (PMC5443548; doi:10.1371/journal.pone.0178149)
Supplement: S1 Table — a Based on Sleddens, O’Connor, Watson, Hughes, Power, Thijs, De Vries, & Kremers. Development of the Comprehensive General Parenting Questionnaire for caregivers of 5–13 year olds International Journal of Behavioral Nutrition and Physical Activity 2014, 11:15. CGPQ adapted to caregivers of 1–4 year olds by Ester Sleddens, Tom Power, Teresia O’Connor, Sheryl Hughes and Stef Kremers. (DOCX) [file pone.0178149.s001.docx]

**S1. Comprehensive General Parenting Questionnaire (caregivers of 1 to 4 year olds)^a^**

|  | **Nurturance = N** |
| --- | --- |
|  | **Nurturance – Autonomy support = AS** |
| 1 | I encourage my child to be curious and to explore things |
| 2 | I let my child make his/her own choices as long as they are safe |
| 3 | I encourage my child to try things on his/her own before I help |
| 4 | I encourage my child to approach things his/her own way, even if it means more work for me |
|  | **Nurturance – Social rewarding = SR** |
| 5 | I praise my child when he/she does something good |
| 6 | I say something nice to my child as a reward for good behavior |
| 7 | When my child does his/her best, I praise him/her |
| 8 | I tell my child how much I appreciate it when he/she helps me |
| 9 | I praise my child when he/she deserves it |
|  | **Nurturance –Responsiveness = R** |
| 10 | I know exactly when things are not going very well for my child |
| 11 | When my child is sad, I know what is going on with him/her |
| 12 | I feel good about the relationship I have with my child |
| 13 | My child and I have warm affectionate moments together |
| 14 | I know exactly when my child has difficulty with something |
|  | **Nurturance – Involvement = I** |
| 15 | I find time to play with my child |
| 16 | I spend a lot of time with my child |
| 17 | I easily find a way to make time for my child |
| 18 | I find it interesting and educational to be with my child for long periods |
|  | **Structure = S** |
|  | **Structure – Inconsistent Discipline = ID** (reverse coded) |
| 19 | I have a hard time consistently enforcing rules with my child |
| 20 | I do not always follow through when I threaten to discipline my child |
| 21 | I threaten discipline more often than I actually give it |
| 22 | There are times I just do not have energy to make my child behave as he/she should |
|  | **Structure – Consistency = C** |
| 23 | When I tell my child I will do something, I do it |
| 24 | I use clear and consistent messages when I tell my child to do something |
| 25 | I try not to change the rules at home very often |
| 26 | I try not to forget the promises I make to my child |
|  | **Structure – Organization = O** |
| 27 | I make sure my child has enough time to get ready for activities |
| 28 | I try to make sure that my child has a regular schedule from day to day |
| 29 | I organize my child’s week so that it follows a regular, predictable pattern |
| 30 | I encourage my child to pick up his/her toys |
|  | I make sure my child is at activities on time |
|  | **Structure – Scaffolding = SC** |
| 31 | When my child is struggling with something, I try to find ways to help him/her |
| 32 | I put time and energy into helping my child, when he/she needs it |
| 33 | When my child has difficulties, I help him/her |
| 34 | When my child has a problem, I help him/her figure out what to do about it |
|  | **Behavioral Control = BC** |
|  | **Behavioral Control – Monitoring = M** |
| 35 | I pay close attention to where my child is |
| 36 | I watch my child to make sure he/she behaves appropriately |
| 37 | I am aware of what my child is doing when he/she is at home |
| 38 | When my child and I are at home together, I frequently check on what he/she is doing |
| 39 | I make sure I know where my child is at all times |
|  | **Behavioral Control – Maturity Demands = MD** |
| 40 | I expect my child to follow our family rules |
| 41 | I have clear expectations for how my child should behave |
| 42 | I require my child to behave in certain ways |
| 43 | I make sure that my child understands what I expect of him/her |
| 44 | I teach my child to follow rules |
|  | **Behavioral Control – Non-Intrusive Discipline = NID** |
| 45 | When I correct my child’s behavior, I explain why |
| 46 | I correct my child when he/she breaks the rules |
| 47 | When my child misbehaves, I point out what he/she did wrong |
|  | **Inappropriate control = CC (Coercive Control)** |
|  | **Inappropriate control – Psychological control (PC)** |
| 48 | When my child does something that is not allowed, I do not talk to him/her for a while |
| 49 | I tell my child I am very disappointed, when he/she does not act appropriately |
| 50 | I make sure my child knows everything I do for him/her |
| 51 | I make my child feel bad when he/she does not meet my expectations |
| 52 | When my child hurts my feelings, I stop talking to him/her until he/she pleases me again |
|  | **Inappropriate control – Physical punishment (PP)** |
| 53 | I spank my child when he/she does not obey rules |
| 54 | I spank my child when he/she does something wrong |
| 55 | I spank my child when he/she is disobedient |
| 56 | I use physical punishment to discipline my child |
| 57 | I spank my child when he/she is behaving inappropriately |
| 58 | **Inappropriate control – Authoritarian control (AC)** |
| 59 | I let my child know that I am the boss in our house |
| 60 | I do not allow my child to get angry with me |
| 61 | I want my child to always obey me |
| 62 | I place a lot of emphasis on obedience in my child |
|  | **Overprotection – OP** |
|  | **Overprotection – Excessive involvement (EI)** |
| 63 | Every free minute I have I spend with my child |
| 64 | I always help my child with everything he/she does |
| 65 | When my child cannot find something, I stop what I am doing to find it before he/she gets too upset |
| 66 | I do not let my child get involved in activities or tasks where he/she might get hurt |
| 67 | I carefully plan my child’s day so that he/she has enough activities to keep him/her busy |
| 68 | I do not let my child stay with our family or friends without me or my spouse present |
| 69 | I always choose what toy my child should play with |

^a^ Based on Sleddens, O’Connor, Watson, Hughes, Power, Thijs, De Vries, & Kremers. Development of the Comprehensive General Parenting Questionnaire for caregivers of 5-13 year olds International Journal of Behavioral Nutrition and Physical Activity 2014, 11:15. CGPQ adapted to caregivers of 1-4 year olds by Ester Sleddens, Tom Power, Teresia O’Connor, Sheryl Hughes and Stef Kremers.
